# Working Remotely, Working Effectively: Improving Collection Access During a Global Pandemic

**DOI:** 10.1177/1550190620980735

**Published:** 2021-06

**Authors:** Colleen Bradley-Sanders

**Affiliations:** 1 Brooklyn College, NY, USA

**Keywords:** working remotely, staff and volunteers, archives, subject focus, case study, access, activities, collections, COVID-19

## Abstract

This article looks at how one college archive responded to the shutdown of its campus in response to the COVID-19 pandemic. The Archivist and Associate Archivist worked together to develop work assignments that could be done from home. While collection processing was halted, the tasks assigned to staff all aimed to improve informational access to the collections, through an expanded effort to convert PDF finding aids to EAD for placement in an ArchivesSpace site, a project to create a searchable listing of collections that includes a brief description of content and links to finding aids, and planning for digitization of frequently accessed content. The archive anticipates having plenty of work to keep staff working even if the campus shutdown continues in the spring, and to date has not had to cut any staff member.

## Introduction

This article examines how the Brooklyn College Archives and Special Collections unit sought to expand access to collection information and also provide meaningful work for full and part-time staff stuck at home during New York state’s “PAUSE” from mid-March 2020 into the mid-summer as the state battled the COVID-19 pandemic. As New York gradually reopened, New York City was the last region to ease the most restrictive elements of the shutdown. Despite the easing of constraints on movement and assembly as the fall 2020 semester got underway, the college remains largely closed to faculty, staff, and students. The archives’ supervisor had to overcome some technology issues, deal with problems faced by staff members at home, and develop a plan for continued work assignments once the library director announced in early August that the archives was one of several units that would not be reopening for the fall semester.

## Background

Brooklyn College, which opened in 1930 as part of the City University of New York system (CUNY), has one of the most diverse student bodies in the country, and the borough in which it resides (Brooklyn) is equally diverse. It is a commuter college, with a large percentage of the students, many of whom are immigrants, the first in their families to attend college.

As of 1979, the college library had a Special Collections division, but no formal archive. Over the next decade there were efforts to begin collecting records of the college, and in 1987 the first professionally trained archivist was hired. Today the archives team is small: full-time staff consists of two archivists, a conservator and a processor, and the number of part-time processors varies from two to four. There is a conservation lab, but no off-site storage.

Under a formal collection development policy instituted in 2018, the archives seeks primarily to document the history of the college, prominent faculty and alumni, and the borough of Brooklyn, while also acquiring significant manuscript collections such as the Hank Kaplan Boxing Archive. It maintains a 12,000+ volume rare book collection that includes the Robert L. Hess Collection on Ethiopia and the Horn of Africa and the Stuart Schaar Collection on the Middle East. There is a limited amount of material currently available online.

## History

When the new college archivist arrived at the archives in the fall of 2015, finding aids were still being created in Word, saved as PDFs and uploaded to a CUNY-wide digital repository called Academic Works. The CUNY Academic Works site is an inflexible platform unsuited to archival needs, and the archives has not added finding aids to it since 2017. In 2014 the archives unsuccessfully presented a plan to the library’s administration for implementing the use of Encoded Archival Description (EAD) for the finding aids, but met resistance from the library’s IT department, which had a number of large projects underway at the time.

In 2016, the archives applied for and won a two-year National Historical Publications and Records Commission (NHPRC) processing grant. One of the project deliverables was an EAD finding aid. The archives looked for a solution to the problem of the IT department being unable to host an EAD site. The archives researched options and settled on ArchivesSpace, in part because other CUNY colleges were already using the program. LibraryHost became the home for the ArchivesSpace page, and the first year’s subscription cost came out of funds controlled by the archives. In the spring of 2019, the finding aid for the collection processed with the NHPRC grant was uploaded to LibraryHost.^1^

How to proceed from there? Other than the college archivist, the staff had no EAD training and there were over 500 finding aids to convert. Already short-staffed, the archives could not devote a processors time to learning EAD and then converting the legacy guides. Nor did they have funding to outsource the work. In the fall of 2019 the archives was awarded a Council on Library and Information Resources (CLIR) Recordings-at-Risk grant. The project budget included funds for a part-time project archivist, and the plan of work included the creation of an EAD finding aid for the newly digitized materials and the larger collection from which they came. With a project archivist experienced in using EAD, the archives made a successful plea to the library administration for another part-time staff member, and so the project archivist was also hired specifically to work on converting the legacy finding aids and to develop a procedures manual that would be used by other staff members as they learned EAD.

## COVID-19 and the Closure of the Brooklyn College Campus

On March 11 the Brooklyn College community was notified that all classes would be moved to a remote learning format after a week’s break for faculty to prepare, but the campus, including the library and archives, would remain open. The immediate impact of this decision meant classes from History, Sociology and Urban Archeology that were slated for hands-on sessions using archival records later in the semester would no longer have the experience of physically searching a collection. The library was going to remain open, so the archives initially planned to temporarily halt processing work and focus on digitizing materials for the affected classes. However, events soon changed those plans.

A late-night email from the CUNY Alert system on March 12 notified faculty, staff and students that the campus would be closed on Friday the 13th, due to a student having tested positive for the virus. The campus was thoroughly cleaned, and reopened on Saturday March 14, but on the afternoon of March 15, heeding the calls of public health officials for social distancing, the college president sent a message stating that supervisors should begin making plans for staff to work from home as much as possible, but this did not include library and archives staff. Hours later the president sent another email stating that following the recommendation of the CUNY administration, Brooklyn College would open only for essential personnel on March 16. The library and archives staff were not included in this group. Non-essential staff, faculty and students could come to campus, but very little would be open, including the library. The archives’ two archivists and the conservator each decided to go to campus, in order to retrieve materials for working from home, since the duration of the shutdown was unknown. As it turned out, with the exception of a handful of visits each from the associate archivist and the conservator, no archives staff member has returned to work on campus, and following an August announcement from the library director, the library and most of the campus will remain closed throughout the fall semester. The earliest staff can return to work in person is late spring 2021, and that is by no means certain.

## Working Remotely

When New York Governor Andrew Cuomo essentially shut down the state with the PAUSE order^2^ that went into effect on March 22, the archivist turned to the problem of figuring out what work the archives staff could perform from home that would benefit the archives and its patrons. The archivists and the conservator all had projects that did not require a daily presence on campus, although later in the shutdown each of them had to return to the archives to retrieve additional material for those assignments. The processing staff and the reading room receptionist presented more of a challenge. They could not take collections home.

**Figure 1.**
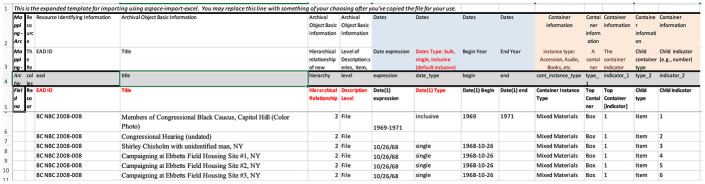
A partial view of the Excel-based template, with entries from the Shirley Chisholm collection.

Increasing the number of finding aids available to patrons online was a long-desired service improvement that would provide patrons with greater information about the collections housed at Brooklyn College. By mid-March, the project archivist, in her other role as a part-time staff member, had converted several finding aids from PDFs to the EAD format. Using an Excel template^3^ that could be uploaded into ArchivesSpace, she simplified the work of inputting the box and folder data for each collection. After some discussion with her, the archivists made the decision to attempt to have the processing staff use the Excel template to do the box and folder data entry for collections, leaving the project archivist to create the individual finding aids in ArchivesSpace. This approach avoided the need to remotely train staff on using the program. It also allowed the archives to offer part-time staffers from other library units some work (Figure 1).

The staff members received the template, instructions, a description of the project, and (for non-archival staff) details of how archival collections are organized before the project archivist conducted a training session via Zoom. The archivist recorded the meeting so everyone could re-watch it as needed, and the project archivist set up “office hours” so people could call with questions if they preferred that to email. There were a few problems. One staff member was so preoccupied with helping her young child cope with distance learning she could not do the assignment. Another lacked a computer. With the college focused on providing laptops for students, there were none for staff, and there were still none seven months later,^4^ nor are there plans to change the situation. The archives purchased a tablet and a case with a built-in keyboard, although it is still not an ideal setup for data entry work. A third employee had difficulty understanding the template and the procedures for the data entry, and so was assigned some online research to assist the associate archivist with the creation of a history timeline for the college. A second Zoom training took place in August, to show the staff how to enter more complex finding aids (more levels of hierarchy in the collection inventories). As the fall semester begins, the finding aid conversion project continues, and should provide meaningful work for the part-time staffers for many months to come. Completed finding aids can be found at https://archives.brooklyn.cuny.edu/repositories/resources. As the summer wore on, it became increasingly unlikely that the campus would reopen for the fall semester, despite the gradual reopening of the state. The library director confirmed this at the August department meeting. While most of the staff could continue working on the conversion of the finding aids, a few needed new assignments.

Many of the collections in the Brooklyn College Archives are simply titled after the donor or creator of the archive, that is, The Papers of Pamella Tucker Farley. The title gives no indication that the papers are those of an activist and important faculty member in the Women’s Studies Program at Brooklyn College. To help patrons better understand what content might be found in a given collection, a staff member has been assigned the task of creating a searchable document that briefly describes each collection. This project would be a much lower priority if the archives were open, yet it will be of value to patrons. The staff member assigned the project is an experienced processor, and would not have been taken away from processing to work on it. When complete, the document will be available on the archives’ web site, and will contain links to existing finding aids on the ArchivesSpace site. Not all finding aids will be converted during the campus shutdown, as there are a fair number of collections that need reprocessing.

**Figures 2.**
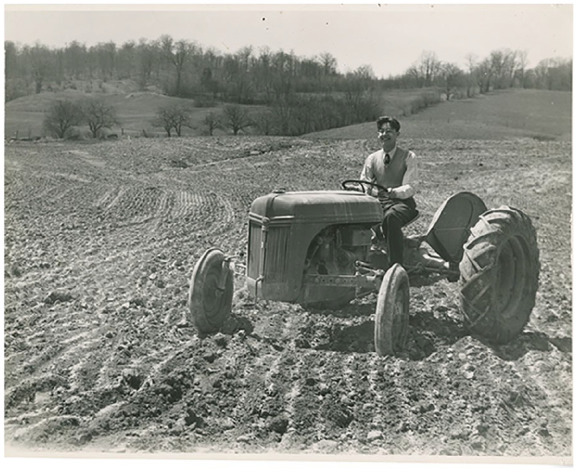
This publicity photo from the archive’s Farm Labor Project collection shows a student driving a tractor (while wearing a tie!). Brooklyn College Archives.

**Figures 3.**
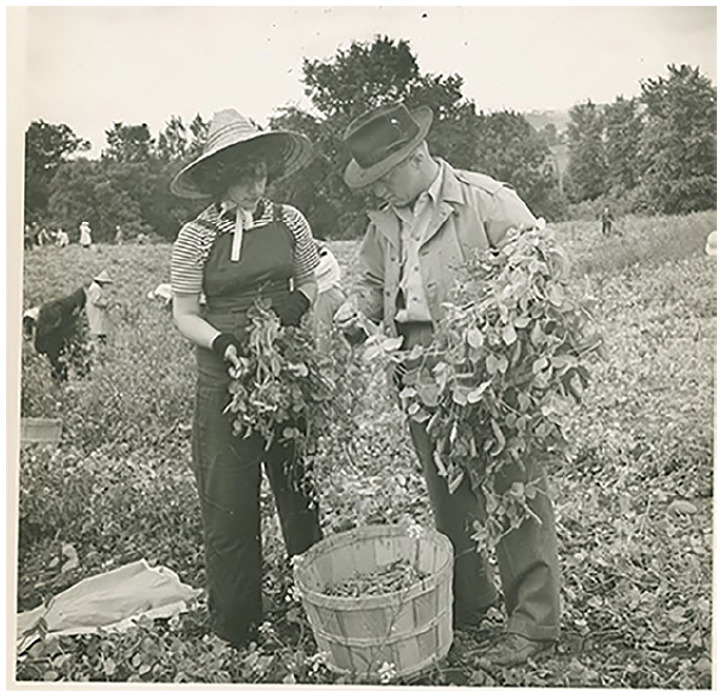
This publicity photo from the archives’ Farm Labor Project collection shows a student learning how to pick peas and beans. Brooklyn College Archives.

Another project to increase access to collections is the digitization of microfilmed student newspapers. The archives has only one functioning microfilm reader, and it can no longer print. There is one reader in the library that can read, print and send digital files. The digitization is being outsourced, and once the digital files are returned, one of the part-time staffers will have the responsibility of uploading the files to the college’s digital platform, on which the archives has its own sub-collection (https://www.njvid.net/showcollection.php?pid=njcore:89131), and also creating the metadata. The student newspaper digitization is part of a larger undertaking to document the more recent history of the college, from the early 1970’s to the present. The archives has significantly less material for this period, and the hundredth anniversary of the college is not far off. Anniversaries generate interest in history, and more requests for information and images from the archives. A related endeavor to digitize and transcribe oral history interviews that are currently inaccessible to patrons (no playback machines) is also underway. The tapes include interviews done as part of the research for a history of the college on the occasion of its fiftieth anniversary in 1980, as well as interviews with students who participated in the World War II-era Farm Labor Project, which was seen as a way for students to participate in the war effort while also having an opportunity to escape the crowded city (Figures 2 and 3). The interviews provide researchers with a different perspective on the program. The college PR was all very positive, but the interviews reveal the students endured poor working conditions, saw harsh and racist treatment of African American workers on the farm, and in at least one case an interviewee remarked that a crop had been dumped in the river once harvested, rather than being sold at market.

## Additional COVID-Related Impacts

Reference service has been brought almost completely to a halt. Research inquiries are piling up, and other than answering a couple of graduation date questions with the help of the Alumni Affairs office, the associate archivist has only been able to answer one research question during one of her infrequent, and brief (three hours), forays to the campus. Time spent in the archives is primarily geared toward gathering materials to enable continued working from home, and until August, there was virtually no light in the archives staff or storage areas as the college sought reduce energy expenditures during the period when only essential personnel were allowed on campus.

Access to campus is restricted and complicated to obtain, and has become more so since the spring. In August, in order to obtain permission for regular, once-a-week access for the associate archivist and the conservator, the archivist had to submit a four-page Access Plan that required the approval of the library director, the college provost, and the college’s COVID Review Board. The Access Plan was rejected in September for the Associate Archivist, but the conservator retains his previously approved weekly access, as he has been designated as essential personnel by virtue of his role on the library’s Disaster Recovery Team. The associate archivist is now unable to work on the backlog of reference questions. The archives’ patrons come from far beyond the confines of the campus. In addition to scholars from other colleges and universities, authors, high school students, and the general public utilize the archives. Between March 13 and September 30, the archives received over thirty-five questions that still need an answer. The most frustrating are those with a deadline, such as photos for a book, or information for a paper.

Fortunately the extended absence from campus has not yet been visibly detrimental to the physical state of the collections. While having almost all lights off completely for several months (which required use of a flashlight when entering the archives) has done no harm, immediately after the shutdown the archivist and conservator emphasized to the library administration the vital importance of someone checking the stacks regularly for leaks. As soon as it was feasible for essential staff to return, either the library director or one of the library IT heads began checking each storage level when they were in the building, and the conservator now does so weekly, along with checking the PEM data recorders on each floor. Surprisingly, there has not been a problem with excessive heat or humidity.

## Conclusion

As the archives looks forward to either a reopening next year with likely restrictions on patron access, or continued working from home, we are making plans to digitize materials frequently used by faculty for their classes, and also materials of high interest, such as yearbooks. Such digitization projects could involve moving scanning equipment to staff member’s homes and require IT staff to coordinate the setup.

While the forced change from the daily routine at the archives has negatively affected our processing of collections and effectively halted any patron access, even via an emailed reference question, the pandemic and subsequent shutdown of the campus have provided an opportunity to step back and think seriously about how and what to digitize to best serve the patrons, to make progress on projects like the conversion of finding aids, and time to think creatively about what else can be done to expand access to the collections at Brooklyn College.

## Footnotes

1. In August 2020, Lyrasis, as part of a CUNY-wide agreement for the hosting of ArchivesSpace sites for individual CUNY schools, became the site host.

2. Link to the New York State Governor’s website and the PAUSE order, available at: https://www.governor.ny.gov/news/governor-cuomo-signs-new-york-state-pause-executive-order (accessed 15 August 2020).

3. The template was downloaded from the Github site, and was one the Project Archivist had used on a previous job with the New York City Municipal Archives, available at: https://github.com/harvard-library/aspace-import-excel (accessed 21 August 2020).

4. October 2020.

